# Nanostructured MoO_3_ for Efficient Energy and Environmental Catalysis

**DOI:** 10.3390/molecules25010018

**Published:** 2019-12-19

**Authors:** Yuhua Zhu, Yuan Yao, Zhu Luo, Chuanqi Pan, Ji Yang, Yarong Fang, Hongtao Deng, Changxiang Liu, Qi Tan, Fudong Liu, Yanbing Guo

**Affiliations:** 1 Key Laboratory of Pesticide & Chemical Biology of Ministry of Education, Institute of Environmental and Applied Chemistry, College of Chemistry, Central China Normal University, Wuhan 430079, China; zhuyuhua@mails.ccnu.edu.cn (Y.Z.); yaoyuan0711@foxmail.com (Y.Y.); luo.z@mail.ccnu.edu.cn (Z.L.); panchuanqi@mails.ccnu.edu.cn (C.P.); yangji@mails.ccnu.edu.cn (J.Y.); fangyarong@mails.ccnu.edu.cn (Y.F.); 2 Department of Civil, Environmental, and Construction Engineering, Catalysis Cluster for Renewable Energy and Chemical Transformations (REACT), NanoScience Technology Center, University of Central Florida, Orlando, FL 32816, USA; chx-liu@knights.ucf.edu (C.L.); qitan@knights.ucf.edu (Q.T.); 3 Key Laboratory of Chemical Utilization of Plant Resources of Nanchang, College of Science, Jiangxi Agricultural University, Nanchang 330045, China

**Keywords:** nanostructured MoO_3_, energy conversion, environmental catalysis, photocatalytic degradation, selective thermocatalysis, water splitting, fuel cells, crystalline structure, morphology

## Abstract

This paper mainly focuses on the application of nanostructured MoO_3_ materials in both energy and environmental catalysis fields. MoO_3_ has wide tunability in bandgap, a unique semiconducting structure, and multiple valence states. Due to the natural advantage, it can be used as a high-activity metal oxide catalyst, can serve as an excellent support material, and provide opportunities to replace noble metal catalysts, thus having broad application prospects in catalysis. Herein, we comprehensively summarize the crystal structure and properties of nanostructured MoO_3_ and highlight the recent significant research advancements in energy and environmental catalysis. Several current challenges and perspective research directions based on nanostructured MoO_3_ are also discussed.

## 1. Introduction

In the 21st century, the impending depletion of fossil fuels and urgent environmental concerns are among the most challenging issues. Exploration of renewable and sustainable energy and the development of new and improved materials are the solutions to keep environmental sustainability [1,2,3,4]. Catalysts are increasingly important in the development of innovative clean energy, environmental protection, and energy conversion [5,6,7]. Accordingly, the search for developing superior nanostructured catalysts is a sustainable alternative. Noble metal catalysts such as Pt, Pd, Rh, and Au exhibit high activity, to tackle the energy and environmental challenges. However, the noble metal catalysts are impacted by their scarcity, high cost, and relatively low stability, which impede their general use in large scale applications. As a result, catalysts with higher activity and lower cost are urgently required for large-scale practical applications [8]. Transitional metal oxides dominate widespread applications in energy and environmental catalysis on account of their relatively low cost, high activity, and stability [9,10,11,12].

Molybdenum oxide (MoO_3_), a kind of transition metal oxide with a n-type semiconducting, nontoxic nature and high stability, has attracted a lot of attention. In particular, nanostructured MoO_3_ has demonstrated superior properties to bulk MoO_3_, which is successfully employed in rechargeable batteries [13], capacitors [14], photocatalysis [15], electrocatalysis [16], gas sensors [17], and other applications [6]. The extended tunnels between the MoO_6_ octahedra in MoO_3_ are suitable for insert/de-insert mobile ions, such as H^+^ and Li^+^, and multiple oxidation states can enable rich redox reactions. Moreover, the superiorities of low cost, chemical stability, high theoretical specific capacity (1117 mA·h/g), and the environmentally friendly nature make nanostructured MoO_3_ exceptional electrode materials for rechargeable batteries capacitors [13,18]. MoO_3_ has been investigated as the photocatalyst in terms of its anisotropic layered structure for absorbing UV, as well as visible light. Introducing a defect band by H^+^ intercalation or oxygen vacancies can create defect state and decrease MoO_3_ bandgap, which effectively increase the photocatalytic activity [15]. Each oxygen atom bonds to only one molybdenum atom of MoO_6_ octahedra, and oxygen vacancy generates Mo dangling bond. MoO_3_ favors the adsorption of water molecules in oxygen vacancies, which act as electron acceptors and consequently reduce the energy barrier make it highly reactive for electrocatalysis [2,19]. As a good gasochromic material, MoO_3_ has efficient positive-ion accommodation and good charge transfer, and it can be used as an optical-based gas sensor. Moreover, relying on the change in the conductance of the oxide-on-gas adsorption/reaction, MoO_3_ has been used for NO, NO_2_, CO, H_2_, NH_3_, and other gases [17]. The unique structure strongly affects the performances. Large efforts have been made to obtain nanostructured MoO_3_ with appealing properties by engineering multiple synthetic strategies for the applications in various fields. A variety of methods have been developed, including hydrothermal method [20], vapor deposition method [21], sol-gel method [22], electrochemical deposition [23], exfoliation method [24], and so forth [25]. Furthermore, many strategies have been used to improve the performance of nanostructured MoO_3_ by controlling compositions, crystal facets, structural blocks, and defects, as well as tailoring the interfacial and electronic structures through doping and hybridization.

Though many of the literature studies have reported on nanostructured MoO_3_, a thorough overview of nanostructured MoO_3_ for efficient energy and environmental catalysis is still lacking so far. Hence, in this paper, we present a comprehensive review of the important advances in nanostructured MoO_3_ materials. We comprehensively introduce the crystalline structures and mainly focus on summarizing the exploitation of nanostructured MoO_3_ in environmental catalysis and energy conversion. We also provide a brief perspective on the current challenges and opportunities for effectively utilizing nanostructured MoO_3_ and taking full advantage of MoO_3_ in constructing highly efficient materials.

## 2. Phase Structure and Morphology of Nanostructured MoO_3_

### 2.1. The Phase Structure of MoO_3_

**Figure 1:**
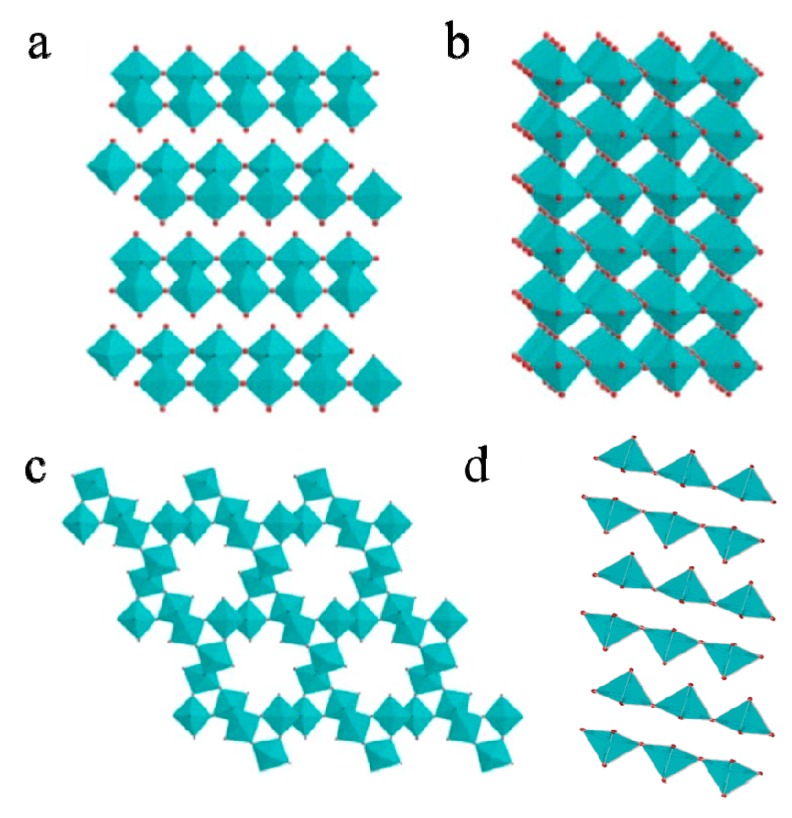
Atomic structures of different crystalline phases of MoO_3_. (**a**) α-MoO_3_, (**b**) β-MoO_3_, (**c**) h-MoO_3_, and (**d**) MoO_3_-II. Reprinted with permission from [6]. Copyright 2018 American Chemical Society.

MoO_3_ involves four different polymorphs (Figure 1), namely the thermodynamically stable orthorhombic phase (α-MoO_3_), the metastable monoclinic phase (β-MoO_3_), the hexagonal phase (h-MoO_3_), and the high-pressure monoclinic (MoO_3_-II) [6,20,26,27]. Each polymorph displays very unique physical and chemical properties, including bandgap energies, refractive indices, and mechanical hardness [28].

Among them, α-MoO_3_ has been extensively studied because of its outstanding electrochemical and catalytic activities. α-MoO_3_ phase is constituted by corner-sharing [MoO_6_] octahedra along [001] and [100] directions. Two sublayers are stacked together by sharing the edges of octahedral along the [001] direction, to form a layer with the orthorhombic symmetry (a = 3.963 Å, b = 13.86 Å, c = 3.696 Å). Furthermore, an anisotropic structure with layers along the [010] direction (b axis) with weak van der Waals forces leads to the formation of α-MoO_3_ (see Figure 1a). The layered structure of α-MoO_3_ has relatively higher tolerance to nonstoichiometry such as the unusual pentavalent ion Mo^5+^, which exhibits high affinity for oxygen, and the layer structure allows the small guest ions (such as Li^+^, H^+^) to be well accommodated, without structural change in principle [20,29,30]. Mai et al. [31] proved that the conductivity and electroactivity of lithiated MoO_3_ nanobelt were highly improved compared to that of a non-lithiated MoO_3_ nanobelt.

The monoclinic structure of β-MoO_3_ is markedly different from the crystal structure of α-MoO_3_, which possesses a ReO_3_-related structure. The MoO_6_ octahedral units share corner oxygen atoms in the direction of the c axis, and edge sharing occurs in the direction of the c axis (see Figure 1b). A transformation from the β to α phase took place spontaneously at the temperature ranging from 387 to 450 °C, according to the reported result. Moreover, β-MoO_3_ exhibited higher catalytic properties than α-MoO_3_ in some catalysis reactions [32,33,34].

Hexagonal h-MoO_3_ is built up by zigzag chains of MoO_6_ octahedra linked to each other by corner sharing along the c axis. A very salient crystalline structure for h-MoO_3_ is the presence of a tunnel (~ 3.0 Å in diameter) running along the c direction. It can serve as a conduit and intercalation site for mobile ions (see Figure 1c). The h-MoO_3_ phase can generally be formulated as (A_2_O)_x_·MoO_3_·(H_2_O)_y_, where A represents an alkali-metal ion or ammonium ion, and the exact values of x and y depend on the details of the preparation and subsequent treatment [6,26,32,35]. The tunnel structure in h-MoO_3_ exhibits considerably higher sensitivity, coloration efficiency, and faster response in comparison with the orthorhombic α-MoO_3_. It is mainly attributed to the h-MoO_3_ of tunnel structure with a higher structure openness degree, giving rise to an accelerated electron–hole separation in photochromism and a facile Li^+^ ion insertion/extraction in electrochromism [36].

High pressure modification of molybdenum trioxide, MoO_3_-II (ε-MoO_3_), the structure of MoO_3_-II is monoclinic, P2_l/*m*_, with unit cell parameters: a = 3.954(l) Å, b = 3.687(2) Å, c = 7.095(4) Å (see Figure 1d). Like the α-MoO_3_ layered structure, the individual MoO_3/3_O_2/2_O_1/1_ layers of MoO_3_-II and α-MoO_3_ are virtually identical. The stacking sequence of the layers of MoO_3_-II (aaa) differs from that of α-MoO_3_ (aba), which is equated with an improved packing efficiency for the layers of MoO_3_-II versus those of α-MoO_3_. The metastable MoO_3_-II can convert rapidly to stable orthorhombic phase α-MoO_3_ at temperatures above 200 °C, which is relatively more stable than h-MoO_3_ [27,28].

The above four different crystal structures have their unique properties, which accordingly influence their applications. The crystal structures of α-MoO_3_ and MoO_3_-II have distinctive double layers. Some positive ions can easily inject into the layer structure, which can be applied in electrochromism, catalysis, and as an electrode material of lithium ion batteries. However, MoO_3_-II is metastable, the crystal structure is easily converted to stable α-MoO_3_, which limits its application. The monoclinic structure of β-MoO_3_ is a monoclinic ReO_3_-related structure, in which each MoO_6_ octahedral shares all the corners with adjacent MoO_6_ octahedral. Breaking the Mo–O bonds can produce more unsaturated Mo atoms on the surface compared with α-MoO_3_, which would behave as active centers for oxidation of small organic molecules, such as partial oxidation of methanol [34]. Unlike α-MoO_3_, metastable h-MoO_3_ easily permits the ready intercalation and migration of some monovalent cations because of the open structure tunnels. The unique structure would play an important role in enhancing the charge transfer characteristics and displaying an ionic conductive nature. The h-MoO_3_ has the potential to exhibit excellent photochromic, electrochromic, and electrochemical properties. Crystal structure is the intrinsic property, but all the stoichiometric MoO_3_ phases have wide bandgaps, which seems to impact some applications. Creating oxygen vacancies, reducing crystal dimensions, introducing dopants, or transforming into quantum dots can be used to manipulate the band structure and improve the performance of MoO_3_.

### 2.2. The Morphology of Nanostructured MoO_3_

A large number of architectures with controllable sizes or morphologies have been reported to optimize the structure and composition, such as zero-dimensional (0D) quantum dots (QDs), and one-dimensional (1D), two-dimensional (2D), and three-dimensional (3D) architectures, which is significant for revealing the relationship between structure and performance [6].

**Figure 2:**
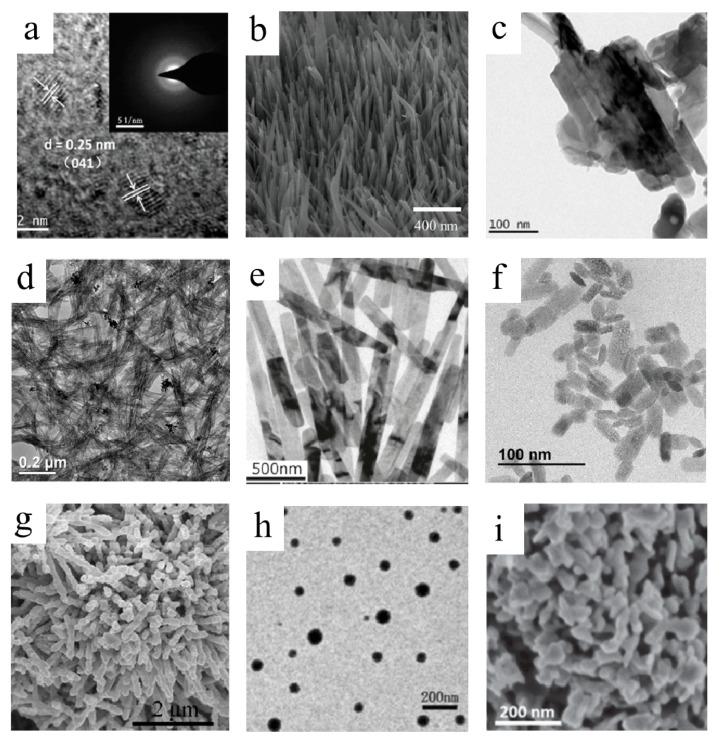
(**a**) HRTEM image of the QDs, the inset shows the SAED pattern of the QDs [39]. Reprinted with permission from [39]. Copyright 2016 The Royal Society of Chemistry. (**b**) SEM image of MoO_3−x_ nanowire array [40]. Reprinted with permission from [40]. Copyright 2012 American Chemical Society. (**c**) TEM image of α-MoO_3_ nanorods [41]. Reprinted with permission from [41]. Copyright 2018 Elsevier Ltd. and Techna Group S.r.l. (**d**) Morphology of the obtained single-walled MoO_3_ nanotubes [42]. Reprinted with permission from ref 42. Copyright 2008 American Chemical Society. (**e**) TEM image of as-prepared h-MoO_3_ nanobelts [26]. Reprinted with permission from [26]. Copyright 2009 American Chemical Society. (**f**) TEM image of MoO_3_ nanosheets [48]. Reprinted with permission from [48]. Copyright 2016 The Royal Society of Chemistry. (**g**) SEM image of MoO_3_ nanoflowers [50]. Reprinted with permission from [50]. Copyright 2014 Elsevier B.V. (**h**) SEM image of MoO_3_ nanospheres [53]. Reprinted with permission from [53]. Copyright 2007 Elsevier B.V. (**i**) SEM image of o mesoporous MoO_3−x_ [55]. Reprinted with permission from [55]. Copyright 2016 WILEY-VCH Verlag GmbH & Co. KGaA, Weinheim.

QDs represent small-size nanocrystals with all three dimensions in several nanometers. The properties were changed significantly from those bulk semiconductors due to their small sizes and the quantum confinement effect [37]. Lu et al. [38] prepared MoO_3_ QDs through combining intercalation and thermal exfoliation and studied the optical properties of MoO_3_ QDs. The dispersion of MoO_3_ QDs showed a tunable strong localized surface plasmon resonance (LSPR) after simulated solar light illumination and the plasmon peak red shifted with an extension of illumination time, which was different from the MoO_3_ nanosheets. Lu and co-workers [39] also observed the morphology changes in combination with different photochromic phenomena through preparation of quantum dots from MoO_3_ nanosheets by UV irradiation (see Figure 2a). It suggested that the morphology changes were mainly influenced by consumption of photoexcited holes.

One-dimensional structure is mainly used to describe structures of which the growth is along one direction and the one dimension is less than 100 nm. One-dimensional architectures can be divided into nanowires, nanorods, nanotubes, nanobelts, and so on [26,40,41,42,43,44,45], which are widely used in many fields due to their unique structural traits. For instance, Meduri et al. [40] reported a MoO_3−x_ nanowire array (see Figure 2b). The 1D MoO_3_ nanowires could provide good conduction pathways for electronic conductivity, along with a shorter path for lithium diffusion, which showed lower lithium intercalation voltages and flat voltage plateau. One dimensional α-MoO_3_ nanorods were prepared with a size of about 50 nm in diameter and few microns long by solution combustion method; the TEM image of α-MoO_3_ nanorods is shown in Figure 2c. By comparison and analysis, the thermodynamically stable α-MoO_3_ single phase exhibited high specific capacitance and good cycle stability for supercapacitor applications [41]. Single-walled MoO_3_ nanotubes were synthesized by Wang and co-workers, and the MoO_3_ SWNTs (see Figure 2d) were synthesized by a thiol-assisted hydrothermal method through an interface controlled self-rolling mechanism [42]. Enyashin et al. [43] proposed the atomic models of MoO_3_ nanotubes and studied the electronic structure and chemical-bonding indices. The results showed that the holey wall structures of MoO_3_ nanotubes made it possible to dope the materials with various atoms. Moreover, α-MoO_3_ nanobelts can display excellent performance for supercapacitors. It was reported that the α-MoO_3_ nanobelts’ electrode exhibited a higher specific capacitance of 369 F/g and a good cycle stability, with more than 95% of the initial specific capacitance maintained after 500 cycles, which suggested the α-MoO_3_ nanobelts could be a potential electrode material [44]. Zheng et al. [26] prepared h-MoO_3_ nanobelts and studied the photochromic and electrochromic properties; the TEM image of as-prepared h-MoO_3_ nanobelts is shown in Figure 2e. Higher structure openness degree in the tunnel structure of h-MoO_3_ could promote electron–hole separation and allow cation insertion/extraction and diffusion which exhibited a better performance on photochromic and electrochromic response than that of α-MoO_3_.

Two-dimensional nanostructured MoO_3_ attracted extensive attention due to its tunable bandgap and high charge carrier mobility. Balendhran and co-workers presented a 2D MoO_3_ based biosensing platform. The nanostructured film made of 2D α-MoO_3_ nanoflakes was used as the conduction channel, which significantly reduced response time due to the high permittivity of the 2D α-MoO_3_ nanoflakes [46]. In addition, Balendhran et al. [47] also demonstrated that the energy bandgap of 2D high-dielectric α-MoO_3_. The α-MoO_3_ forming MoO_3−x_ can reduce the bandgap and enhance charge carrier mobility. Compared with the bulk MoO_3_, 2D-MoO_3_ nanosheets had a better chemical sensor performance due to the 2D structure with a large surface area and more reactive sites [48] (see Figure 2f). Cheng et al. [49] reported that MoO_3−x_ nanosheets can display strong LSPR from the visible to the near-infrared region, owing to the layered crystal structure, which could be used as highly efficient catalysts for the hydrogen generation from ammonia borane with visible light irradiation.

Three-dimensional architectures can increase the surface area and provide more facets with active sites. Three-dimensional architectures mainly include nanoflowers, nanospheres, and mesoporous structures. Nanoflowers can be described as compositions of low-dimensional nano building blocks for certain structures, such as nanorods, nanosheets, and so forth. Sui et al. [50] reported a flower-like α-MoO_3_ with hierarchical structure (see Figure 2g). The sensors based on α-MoO_3_ flowers exhibited highly sensitive and distinctively selective to trimethylamine. The α-MoO_3_ flowers may have less agglomerated configurations, more effective charge transportation, and expose more active sites, as well as specific facets. Another type of promising architecture, nanospheres were widely used in the fields of catalysis, sensing energy storage conversion, and so on [51]. Generally, hollow MoO_3_ nanospheres were prepared by drawing support from templates. Triblock copolymer micelles have been used as templates to form hollow MoO_3_ nanospheres. The size could be adjusted by choosing a suitable micellar core [52]. Du et al. [53] synthesized highly dispersed MoO_3_ nanospheres by ultrasonic irradiation (see Figure 2h) and probed their growth mechanism. Mesoporous structure because of their intrinsic high surface areas and the nano-wall structure were beneficial to diffusing the ions and electrons, which can enhance the electrochemical or photocatalytic properties [6]. Brezesinski and co-workers reported the ordered mesoporous α-MoO_3_ nanocrystalline walls for the application of thin-film pseudocapacitors, which exhibited the superior capacitive charge-storage properties compared with both the mesoporous amorphous and nonporous crystalline MoO_3_ [54]. Luo et al. also prepared mesoporous MoO_3−x_ (see Figure 2i) with good activity and stability for HER under both acidic and alkaline conditions [55].

Based on the above overview, the performance of MoO_3_ is generally influenced by the morphology, and the essence is that the structure of MoO_3_ is different. The controllable preparation of diversified nanostructured MoO_3_ is an efficient approach to achieve excellent performance. However, it should be noted that some nanostructured MoO_3_ with well-controlled morphologies and sizes were not applied well. Hence, it is better to rationally design and synthesize with well-controlled nanostructured MoO_3_ based on advances in nanomaterials science and engineering.

## 3. The Application of MoO_3_ in Energy Related Catalysis

Due to its low-cost, nontoxicity, multiple oxidation states, and van der Waals gap along the [010] direction, molybdenum oxide (MoO_3_) has attracted more and more attention in hydrogen evolution, oxygen evolution, and fuel cells in recent years. However, because of its poor inherent electrical conductivity and slow electrochemical kinetic process, the widespread application of MoO_3_ is limited, and a great amount of effort has been devoted to enhancing the catalytic performance of MoO_3_.

### 3.1. Application in Hydrogen Evolution

Owing to high calorific value and its renewable and clean properties, hydrogen (H_2_) is regarded as one of the most promising energy carriers for the future. As a layered n-type semiconductor, MoO_3_ has been studied as a catalyst for electrocatalytic hydrogen evolution, photocatalytic hydrogen evolution, and ammonia borane dehydrogenation.

#### 3.1.1. Electrocatalytic Hydrogen Evolution Reactions

**Figure 3:**
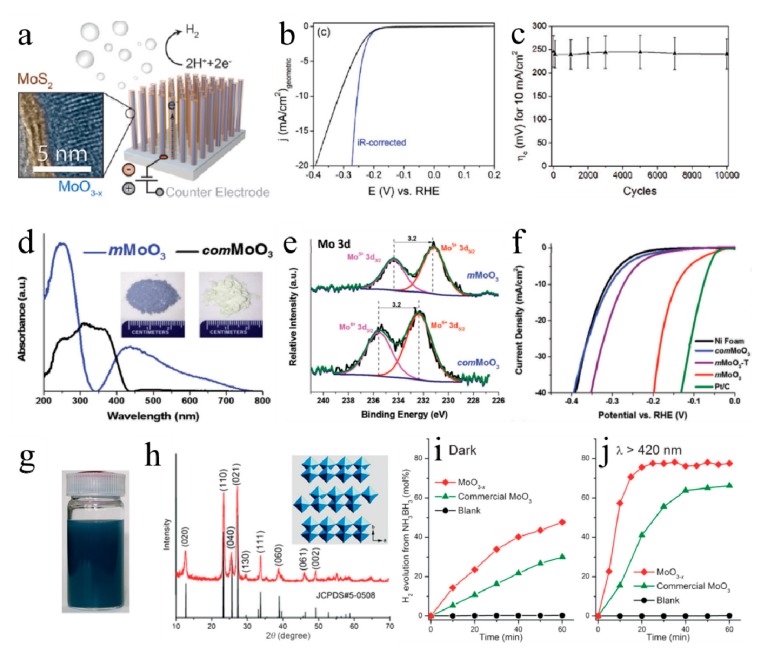
(**a**) Schematic diagram and TEM image of core–shell MoO_3_–MoS_2_ nanowires applied in hydrogen evolution; (**b**) the HER activity of the nanowires sulfidized at 200 °C is presented with its iR-corrected data; (**c**) the cycling stability of nanowires sulfidized at 200 °C, measured as current density at –0.4 V vs. RHE, normalized to initial current density [56]. Reprinted with permission from [56]. Copyright 2019 American Chemical Society. (**d**) Diffuse reflectance ultraviolet-visible spectra (DR UV-vis) and photos (onset images) for *com*MoO_3_ and as-synthesized *m*MoO_3_; (**e**) XPS spectrum details for Mo 3d binding energy regions; (**f**) polarization curves of *m*MoO_3_ materials on Ni foam electrode in 0.1 M of KOH [55]. Reprinted with permission from [55]. Copyright 2016 WILEY-VCH. (**g**) A photograph of the MoO_3−x_ product dispersed in ethanol; (**h**) XRD pattern of MoO_3−x_ and (inset) crystal structure of the orthorhombic MoO_3_. Time course of H_2_ evolution from NH_3_BH_3_ aqueous solution at room temperature for different samples: (**i**) in the dark and (**j**) under visible light irradiation (λ > 420 nm) [49]. Reprinted with permission from [49]. Copyright 2014 WILEY-VCH.

Electrocatalytic hydrogen evolution reaction (HER) is an efficient method. It is an environmentally friendly process that does not create any by-products. Although the layered structure of MoO_3_ is suitable for insertion/removal of small ions such as H^+^, MoO_3_ always shows poor catalytic performance for HER, due to the lack of active sites [55]. Therefore, MoO_3_ always serves as core the substance, as the MoO_3_/MoS_2_ core–shell structure [56,57,58]. The MoO_3_ core provides high specific surface area and facile charge transport, as depicted in Figure 3a. In order to eliminate the influence of the resistance of the solution and obtain the real kinetic activity of the MoO_3_/MoS_2_, an iR-corrected test was also performed, and the data is shown in Figure 3b. The HER activity of MoO_3_/MoS_2_ remained stable even if it was tested for 10,000 cycles (Figure 3c) [56]. Besides, Pt [59], Pd [60], RuO_2_ [19] can also load on MoO_3_, to get excellent electrocatalysts for electrocatalytic hydrogen evolution.

In order to extend application in electrochemical hydrogen evolution, researchers found that oxygen vacancies can dramatically improve catalytic performance of MoO_3_. Figure 3d showed that MoO_3_ with oxygen vacancies possessed a much narrower band gap compared to commercial MoO_3_, and the color changed from greenish to blue (Figure 3d onset). Figure 3e demonstrates the formation of oxygen vacancies in MoO_3_. As shown in Figure 3f, the oxygen vacancies which were close to Mo^5+^ can serve as active sites [55]. In this way, 2D α-MoO_3_ nanosheets were fabricated and exhibited a considerable HER performance with low overpotential of 142 mV, to achieve 10 mA/cm^2^ current density [61]. Zhang et al. also found that MoO_3−y_ (valence state of V and VI) can be used for efficient electrocatalytic hydrogen evolution [62]. Second, the morphology is another key factor. Mesoporous MoO_3_ [55] and MoO_3_ nanosheets [61] mentioned above have demonstrated the importance of morphology. Zhang et al. synthesized porous MoO_3_ with large specific surface area of 113.8 m^2^/g, and it can work as a bifunctional electrocatalyst for (oxygen evolution reactions) OER and HER [63]. The large specific surface area of as-synthesized porous MoO_3_ increased electrochemical active area (0.057 mF/cm^2^) which was four times bigger than commercial MoO_3_ (0.012 mF/cm^2^). Third, hetero-atoms doping is another efficient strategy to improve electrocatalytic performance of MoO_3_. Li et al. have confirmed that P doped MoO_3−x_ nanosheet (P–MoO_3−x_) can show the low overpotential of 161 mV to reach 10 mA/cm^2^ current density with low Tafel slope of 42 mV per decade [64]. They also found that P doping sites could attract protons to adjacent oxygen vacancies and then form H_ads_ on oxygen vacancies, which meant that P doping sites and oxygen vacancies promoted HER together. Haque et al. also demonstrated that NH_4_^+^ doped MoO_3_ (2D Crys–AMO) just need 138 mV to achieve 10 mA/cm^2^ current density. After NH_4_^+^ doping, orthorhombic MoO_3_ was transformed into hexagonal phase, resulting in the formation of highly ordered intracrystalline pores (serve as active sites) [65]. When the hetero-atoms doping leads to the structure defect, such as oxygen vacancies or transformation of intracrystalline phase, the HER activity of MoO_3_ may be promoted dramatically. In addition, when the atomic radius of hetero-atoms is similar to oxygen, it may be easier to be doped into the lattice of MoO_3_. Stability is another crucial factor for catalysts. In general, MoO_3_-based nanocatalysts, which served as substrate or active center, possessed excellent stability in both acid and alkaline media for HER [19,55]. The current density can remain stable at a specific voltage for 12 [55], 24 [19], and even 40 h [65].

#### 3.1.2. Photocatalytic Hydrogen Evolution Reactions

Because solar energy is inexhaustible, photocatalytic hydrogen evolution reaction is an attractive strategy to produce hydrogen from water [66]. MoO_3_ is a well-known direct-band-gap semiconductor with high work function and good hole conductivity. However, due to wide band gap energy (about 3.0 eV), the photo-induced electrons/holes (e^−^/h^+^) pairs are easy to recombine, resulting in low conversion efficiency of incident light [67]. To solve this issue, MoO_3_-based nanocomposites were synthesized and studied. Ma et al. fabricated an MoO_3_/polyimide composite, and the growth of MoO_3_ increased the light absorption and suppressed the recombination of e^−^/h^+^ pairs [66]. MoO_3_–TiO_2_ nanotubes were studied, and MoO_3_–TiO_2_ annealed at 650 °C showed an almost-100-times-higher donor concentration than pure TiO_2_ nanotubes. The MoO_3_–TiO_2_ nanotubes possessed lower charge transfer resistance and improved separation efficiency because of the appearance of MoO_3_ [68]. Esparza et al. fabricated a Mo-coated Pt HER catalyst which was O_2_-insensitive and stable in acidic media. The formed Mo membrane kept H_2_ and O_2_ far away from Pt, suppressing both oxygen reduction reactions (ORR) and hydrogen oxidation reactions (HOR). Therefore, the HER efficiency of the Mo-coated Pt HER catalyst was promoted [69]. Guo et al. synthesized MoS_2_@MoO_3_ core–shell nanowires with a high hydrogen evolution rate of 841.4 µmol/(h·g). The MoO_3_ widened the range of light absorption and produced more photo-induced carrier, which accelerated HER rate greatly [70]. Direct bonds formed between CdS NWs and MoO_x_ clusters were achieved. The MoO_x_ clusters induced deep electron-trap states by generating long-lived electrons, to improve the activity. The MoO_x_ clusters could also effectually dissociate adsorbed water molecules, resulting in improved photocatalytic HER activity [71]. Under light irradiation, no obvious diminution in the photocatalytic activity of MoO_3_-based materials for HER was observed for 15–30 h [66,67,68,69,70,71].

#### 3.1.3. Ammonia Borane Dehydrogenation

Due to nontoxicity, room-temperature stability, and high hydrogen storage content (19.6 wt.%), ammonia borane (NH_3_BH_3_; AB) has been regarded as an attractive hydrogen storage material candidate [49,72]. With suitable catalysts, the hydrolysis of NH_3_BH_3_ can be obtained as illustrated in Equation (1):

NH_3_BH_3_ + 2H_2_O → NH_4_^+^ + BO_2_^−^ + 3H_2_ (1)

MoO_3_ shows strong LSPR signal, a near-field enhancement phenomenon, in the visible light region. Yamashita group firstly reported visible-light-induced hydrogen evolution enhancement from NH_3_BH_3_ solution in plasmonic MoO_3−x_ [49], and the blue color of MoO_3−x_ (Figure 3g) was consistent with another report [55]. Figure 3h showed that the MoO_3−x_ is orthorhombic phase. The activity of MoO_3−x_ was obviously higher than commercial MoO_3_ under visible light irradiation (Figure 3i,j). After that, they prepared Pd/MoO_3−x_ hybrid and it exhibited great plasmon-enhanced hydrolysis of NH_3_BH_3_ [72]. The MoO_3−x_ nanoparticles were fabricated, and the enhanced LSPR property generated by the introduction of oxygen vacancies [73]. Further, they explored the effect of reduction temperature to MoO_3_, and MoO_3−x_-200 °C (H_2_ reduction temperature) showed a higher dehydrogenation activity [74]. The oxygen vacancies could narrow the band gap of MoO_3_, then increased the absorption of visible light in a wider range. Lu et al. synthesized MoO_3_-doped MnCo_2_O_4_ microspheres comprised of nanosheets. The Mo doping provided a small pore diameter leading to an enhanced specific surface area (BET area changed from 13.2 to 62.1 m^2^/g), which is contributed to enhance hydrolysis of NH_3_BH_3_ [75]. The stability of existing MoO_3_-based catalyst is not desirable due to relatively short test time (60–70 min) [72,73,74,75].

In summary, the application of MoO_3_ in hydrogen evolution is still in preliminary stage. And the further improvement in the activity and stability is still anticipated. Most of works reported that MoO_3_ can serve as substance instead of active center in electrocatalytic and photocatalytic hydrogen evolution. As for NH_3_BH_3_ dehydrogenation, Yamashita group has been devoted to developing MoO_3_ as a promising material. There still exists broad space for the researchers to develop nanostructured MoO_3_ as an excellent catalyst for hydrogen evolution. The possible strategy including introduction of oxygen vacancies, hetero-atom doping and hybridization with other materials.

### 3.2. Application in Oxygen Evolution

#### 3.2.1. Electrocatalytic Oxygen Evolution Reactions

**Figure 4:**
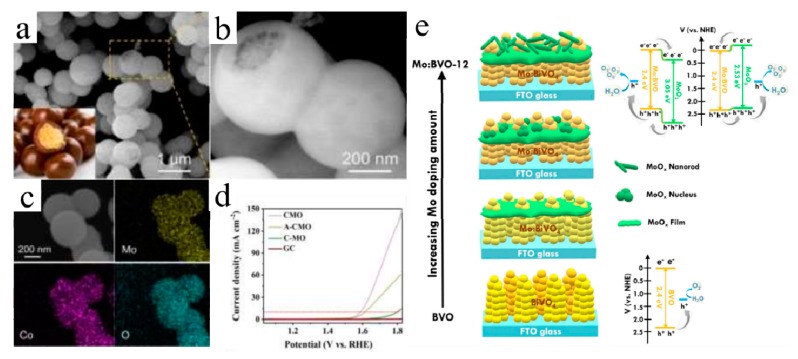
(**a**) SEM image of CoMoOx (inset: the picture of MYLIKES chocolate with unique core–shell structure); (**b**) SEM image of CoMoO_x_ with high magnitude; (**c**) SEM and element mapping images of Mo, Co, and O in CoMoO_x_ nanospheres; (**d**) polarization curves comparison between CoMoO_x_ (CMO), A–CoMoO_x_, and C–MoO_x_ [78]. Reprinted with permission from [78]. Copyright 2019 Royal Society of Chemistry. (**e**) The schematic illustration for the formation of the Mo:BVO/MoO_x_ heterojunction. The band structures along with the charge transfer cascade for the BVO and Mo:BVO-12 electrodes were also shown in the bottom side [82]. Reprinted with permission from [82]. Copyright 2019 Elsevier B.V.

Electrocatalytic OER is another half-reaction of water splitting. Owing to transfer of four electrons for the evolution of an O_2_ molecule, OER is more sluggish compared to HER and becomes the rate-determining step of water splitting [76,77]. There are just a few of research studies about MoO_3_ applied in OER, in which MoO_3_ is regarded to be catalytically inert for OER [78]. Tariq et al. prepared IrO_2_–MoO_3_ composites and 30% mole fraction of iridium contents, which would be more favorable for OER. The mass specific OER activity of iridium active centers was greatly enhanced by seven-fold [76]. NiO@MoO_3_/Vulcan carbon was also investigated by Illathvalappil. The MoO_3_ would react with NiO to form a thin NiMoO_4_ film which could stop MoO_3_ from dissolving in the alkaline media, resulting in excellent stability at 1.51 V (vs. RHE) for 15 h in 1 M of KOH solution [77]. Guo et al. fabricated Co-doped MoO_x_ (CMO) core–shell structure. Figure 4a showed the CMO with uniformly spherical structure of 500 nm in diameter. Further, on a damaged area (Figure 4b), the core–shell structure can be observed, which means that the CMO possesses a MYLIKE chocolate-like structure. And Figure 4c further demonstrated the uniform distribution of Co, Mo, and O in the CMO. The CMO exhibits the overpotential 340 mV to reach 10 mA/cm^2^ current density with a Tafel slope of 49 mV per decade (Figure 4d) [78]. The co-doping, bringing numerous active sites (about 6.550 × 10^−3^ mol/g) and amorphous structure of the CMO, was responsible for the improvement of OER activity. The durability of these catalysts was not so good, and the current density could remain stable at 10 mA/cm^2^ for only 3–15 h in KOH aqueous solution [76,77,78].

#### 3.2.2. Photoelectrochemical Oxygen Evolution Reactions

Photoelectrochemical (PEC) water splitting has been regarded as a potential avenue for sustainable energy supply. A main obstacle is the need of efficient and stable water oxidation photocatalysts [79,80]. As a high work function (>6.3 eV) and layered semiconductor, MoO_3_ always hybridizes with other materials (such as BiVO_4_) in order to match band potential and obtain an efficient catalyst. Lou et al. fabricated a Bi_2_MoO_6_/MoO_3_ heterojunction by using anodic oxidation of a molybdenum foil and, subsequently, a hydrothermal method at 160 °C for 24 h. The Bi_2_MoO_6_/MoO_3_ photoanode showed about 100% faradic photocurrent-to-O_2_ conversion efficiency. They found that, when Bi^3+^ was introduced into the MoO_3_ membrane, the valence band moved upward, which would lengthen the wavelength of light absorbed by Bi_2_MoO_6_/MoO_3_. The photocurrent could remain stable for 8 h by using the Bi_2_MoO_6_/MoO_3_ [79]. He et al. prepared the MoO_3_/BiVO_4_ heterojunction film by a drop-casting method, wherein the BiVO_4_-based precursor was dropped on FTO glass and then dried and annealed. At 0.8 V (vs. SCE), the photocurrent of MoO_3_/BiVO_4_ was six times higher than bare BiVO_4_ film. The improvement can be attributed to band potentials and conductivity differences between MoO_3_ and BiVO_4_ [80]. MoO_3_/Ag/TiO_2_ nanotube arrays were also successfully synthesized and investigated systemically. The enhanced photoelectrochemical performance is related to the tight contact at the interface. Specifically, the photoinduced electrons could move from valence band of TiO_2_ to conduction band of MoO_3_ through a Ti–O–Mo bond [81]. Chen et al. prepared Mo-doped BiVO_4_/MoO_x_ electrode (1.2% Mo), and Figure 4e shows the formation of the Mo-doped BiVO_4_/MoO_x_ heterojunction. With the increase of Mo doping amount, MoO_x_ would gradually accumulate and form a film on the surface of BiVO_4_, and then continue to grow on the film. At 1.23 V (vs. RHE), the photocurrent density of the catalyst (2.67 mA/cm^2^) was five-fold higher than that of BiVO_4_ electrode [82]. The valence and conduction band edges of BiVO_4_ and MoO_x_ are suitable for forming the type II heterojunction at the interface and then accelerate the carrier transfer and separation.

In short summary, the research on MoO_3_ for the application of OER and PEC water splitting is limited, but the published reports indicate that MoO_3_ is a promising OER and PEC catalyst through morphology and structure design. MoO_3_ has a wide band gap (about 3 eV) and easily forms heterojunctions with other materials, so as to obtain photoelectric OER catalysts with higher activity. More effective methods are needed to exploit the potential of MoO_3_. Meanwhile, the reason for enhanced catalytic behavior also needs to be further investigated, to provide a principle for designing next-generation electrocatalysts.

### 3.3. Application in Fuel Cells

#### 3.3.1. Direct Methanol Fuel Cells

**Figure 5:**
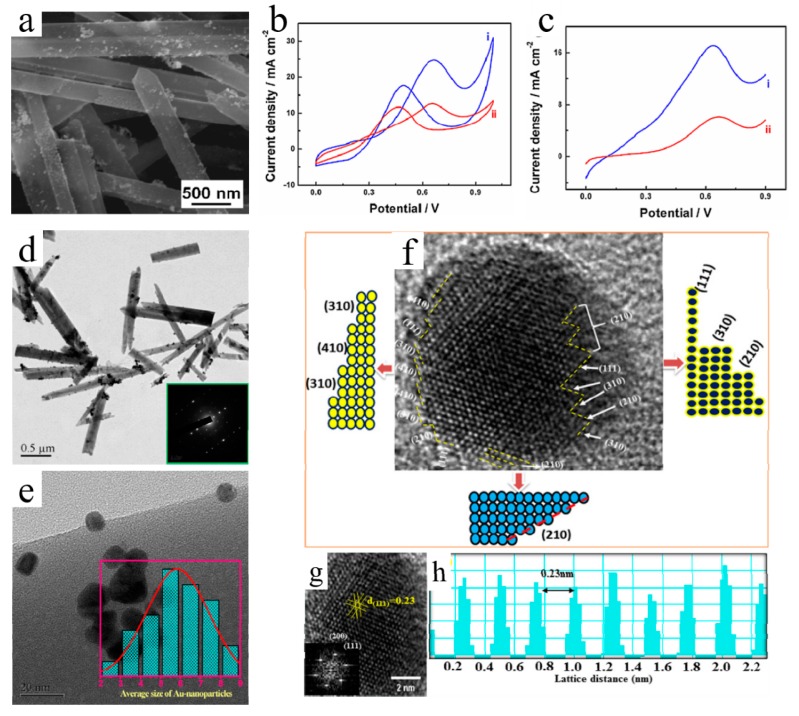
(**a**) FESEM image of the prepared Pt/MoO_3_ nanocatalysts; (**b**) CVs of the (i) Pt/MoO_3_ and Pt/C (ii) catalysts in 0.5 M of H_2_SO_4_ containing 1.0 M of CH_3_OH; (**c**) LSVs of the (i) Pt/MoO_3_ and Pt/C (ii) catalysts in 0.5 M of H_2_SO_4_ containing 1.0 M of CH_3_OH [86]. Reprinted with permission from [86]. Copyright 2015 Elsevier B.V. (**d**) TEM image of Au/MoO_3_ (inset: corresponding SAED pattern); (**e**) HRTEM image of Au/MoO_3_ (inset: the average size of Au nanocrystals calculated from histograms); (**f**) Typical HRTEM lattice image of high index surfaces Au nanocrystals; (**g**) HRTEM image shows the majority of lattice plane (111) facet of Au nanocrystals, the inset in (**g**) indicates FFT pattern of Au nanocrystals enclosed with (111) and (200) plane; (**h**) IFFT profile of Au nanocrystals lattice distance analysis [91]. Reprinted with permission from [91]. Copyright 2017 Elsevier Ltd.

With increasing demand of energy, the direct methanol fuel cells (DMFCs) have attracted more and more attention due to their high energy density (5 kW·h/L). However, the low activity of catalysts and the use of noble metals block its commercial application [83,84,85,86]. Nonstoichiometric MoO_3_ (MoO_x_) has a rutile-type structure and shows metallic conductivity, which is relatively stable and highly active [83]. Cabrera et al. first reported Pt/MoO_x_/glassy carbon used for DMFCs by using an electrochemical deposition method. The lower valence of molybdenum and the proton spillover effect from hydrogen molybdenum bronze may account for the improved activity [83,84]. Justin et al. also prepared Pt–MoO_3_/C composite. Insulating MoO_3_ was electrochemically reduced to conductive hydrogen molybdenum bronze (HxMoO_3_), which can keep the Pt surface clean in order to oxidize methanol. MoO_3_ plays a crucial role in the transition process of adsorption intermediates to carbon dioxide [85]. Zhang et al. fabricated Pt/MoO_3_ nanowires by using an impregnation–calcination method. After loading of Pt nanoparticles, the Pt/MoO_3_ maintained its morphology of nanowires, as shown in Figure 5a. Figure 5b,c shows cyclic voltammetry, linear sweep curves of Pt/MoO_3_ and Pt/C, respectively. The Pt/MoO_3_ showed much higher electrocatalytic activity and stability for the methanol oxidation, compared to Pt/C (the same Pt loading) [86]. The promoted activity may be attributed to more active catalytic sites and three-dimensional structures generating reactant diffusion microchannels. The stability of MoO_3_-based catalysts mentioned above is not satisfactory, and the time of stability test is less than 1 h.

#### 3.3.2. Oxygen-Reduction Reactions

As a type of high efficiency, environmentally friendly energy conversion device, fuel cells have attractive much more attention in recent years [87]. Platinum (Pt) is a benchmark catalyst and always shows excellent performance for ORR. However, it is impossible to use Pt in practical applications that are large in scale because of its scarcity and high cost. Therefore, developing low-content Pt catalysts is a promising way and can be used for real application eventually. Recent researches indicated that MoO_3_ can modify a Pt electrode to enhance its catalytic performance and stability [88,89]. After electrodeposition of MoO_3_, the Pt/MoO_x_/GCE showed a significant increase in the cathodic peak current. The improved performance could be ascribed to the combinations between Pt and MoO_3_, which is a doped effect that always happens between transition elements and leads to the optimal mutual electronic density of states [90]. Karuppasamy et al. fabricated Au–MoO_3_ and efficiently decreased the usage amount of Au. The Au/MoO_3_ exhibited a one-dimensional morphology of nanorods, and there was no obvious aggregation of Au nanoparticles (Figure 5d). Further, according to particle size of statistics, the mean diameter of Au nanoparticles was 5.9 nm (Figure 5e). After 1000 cycles, no obvious change in the half-wave potential was observed, indicating excellent durability. The HRTEM was employed to study the specific structure of Au/MoO_3_. The MoO_3_ made the appearance of low-coordinated stepped Au atoms in the edge, such as (210), (310), and (410), indicating these were main planes of Au nanoparticles (Figure 5f). Then, Figure 5g,h confirmed that the majority of planes of Au nanoparticles was (111) plane. Due to the presence of MoO_3_, the formation of poisonous intermediates, such as OH species, was suppressed on the high index plane of Au nanoparticles; therefore, oxygen was reduced more efficiently [91].

In summary, the glassy carbon electrodes with Pt-modified MoO_3_ have been investigated widely for DMFCs and ORR. The MoO_3_ can modify the electronic structure of Pt and suppress the formation of poisonous intermediates on high index plane of Au. However, MoO_3_ directly used for DMFCs or ORR has not been reported. This phenomenon may be assigned to the relatively fewer research studies and the fact that MoO_3_ always serves as a catalyst of partial oxide, suggesting it does not exist at the active sites for breaking O–O bonds. Besides these points, the oxidation mechanism of methanol and oxygen is complex, and there is no clear mechanism explanation about MoO_3_-based materials for DMFCs and ORR. A lot of work still needs to be done for MoO_3_-based materials applied in DMFCs and ORR.

## 4. MoO_3_ Applied in Environmental Catalysis

### 4.1. Photodegradation of Organic Pollutants

Photocatalysis is the acceleration of a photoreaction rate in the presence of a catalyst, and it is a green and sustainable catalytic technology that has been widely studied for chemical synthesis, water treatment, environmental cleaning, and self-cleansing processes [92]. The photocatalytic reaction occurs at the interface, based on the absorption of photons with energy larger than the band gap of photocatalyst, with electrons excited from the valence band to the conduction band and producing electron–hole pairs. Photocatalytic efficiency mainly depends on the power and wavelength of the photon source; the properties of the catalyst include its electronic structure, defect density, surface area, and surface-to-volume ratio [93]. Therefore, improving the adsorption capacity of organic pollutants and enhancing the ability of light capture and photo-induced generation performance of electron–hole pairs are important. MoO_3_ is one of the most important photocatalysts because of its interesting semiconducting layered structure, rich chemistry associated with multiple valence states and high thermal and chemical stability. Due to the abovementioned outstanding properties, nanostructured MoO_3_ has been extensively explored for photocatalytic processes, which are summarized below.

Organic dyes are widely used in textile manufacturing, but they are an environmental threat; hence, the treatment of dyes in wastewater is highly necessary [94]. In general, the degradation rates of rhodamine B (RhB), methylene orange (MO), and methylene blue (MB) are used to evaluate the photocatalytic activity of catalysts. Owing to the distinctive layered structure, MoO_3_ exhibited strong adsorption performance and good photocatalytic activity for photodegradation organic pollutants [95,96,97,98]. Inadequately, the large band gap (2.7–3.2 eV) of MoO_3_ usually limits the photocatalytic performance of decomposing organic pollutants [99,100]. Based on MoO_3_, various strategies are employed to develop the new efficient photocatalytic materials with the desired properties. These include the doping collaborated with other applicable semiconductors or elements and the fabrication of composites of heterostructures [15].

**Figure 6:**
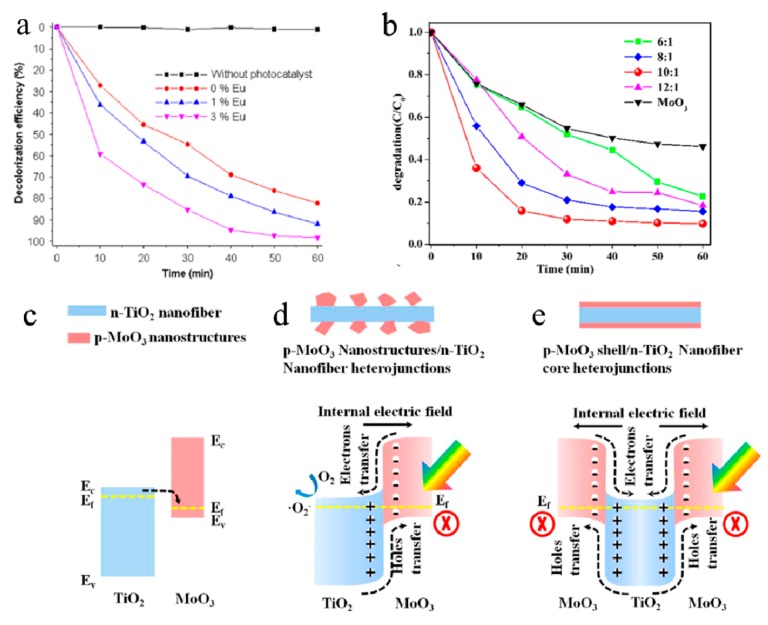
(**a**) Decolorization efficiency of 3 mol% Eu-doped MoO_3_ [101]. Reprinted with permission from ref 101. Copyright 2016 Elsevier. (**b**) Photocatalytic activities in RhB degradation for pure MoO_3_ and MoO_2_/MoO_3_ nanocomposites with different contents of MoO_2_ under visible-light irradiation [100]. Reprinted with permission from [100]. Copyright 2019 Elsevier. (**c**–**e**) The energy band alignment of p-MoO_3_ nanostructures and n-TiO_2_ nanofiber heterojunctions and the postulate mechanism for photodegradation of RhB under UV irradiation [103]. Reprinted with permission from [103]. Copyright 2014 American Chemical Society.

Hybrid nanomaterials can be designed to help enhance the degradation efficiency. Lots of efforts have been made in the design of MoO_3_-based hybrids such as Eu(Gd)-doped MoO_3_ [101,102], rGO/C-MoO_3_ [15], and MoO_2_/MoO_3_ [100]. For instance, Phuruangrat et al. synthesized Gd-doped MoO_3_. The Gd-doped MoO_3_ nanobelts showed better degradation activity of MB than pure MoO_3_, 99% of MB could be degraded by 3 mol% Gd-doped MoO_3_ under visible-light irradiation in 60 min (see Figure 6a). MoO_2_/MoO_3_ hybrid nanostructures were prepared by Xi and co-workers [100]. The photocatalytic activities in RhB degradation for pure MoO_3_ and MoO_2_/MoO_3_ nanocomposites with different contents of MoO_2_ under visible-light irradiation are shown in Figure 6b. MoO_2_/MoO_3_ exhibited high-efficiency degradation for RhB with the assistance of H_2_O_2_. The ·OH was a dominant reactive species for the degradation of RhB under visible-light irradiation, and the synergistic effect between dyes and MoO_2_/MoO_3_ nanoparticles accelerated the production of hydroxyl radical (·OH) from H_2_O_2_ in this system.

Nanocomposites of heterostructures have also been explored to harvest visible-light nanocomposite, and exhibited extremely enhanced visible-light photocatalytic activity for decomposing organic pollutants. Lu et al. [103] fabricated p-MoO_3_ nanostructures/n-TiO_2_ nanofiber heterojunctions. The p-n nanoheterojunctions decreased photoluminescence intensity, suppressed the recombinations of photogenerated electrons and holes, and enhanced charge separation and photocatalytic efficiencies. Figure 6c–e showed the energy band alignment of p-MoO_3_ nanostructures and n-TiO_2_ nanofiber heterojunctions and the postulate mechanism for photodegradation of RhB under UV irradiation. Nanostructures/n-TiO_2_ nanofiber heterojunctions exhibited a two-times-higher first-order rate constant for the degradation of RhB than that of TiO_2_ nanofibers. Significant improvement of the visible-light-driven photodegradation has also been observed, using Bi_2_Mo_3_O_12_/MoO_3_ [104], MoS_2_/MoO_3_ [105,106], and TiO_2_/MoO_3_ heterostructures [107]. It was attributed to combining a feasible semiconducting material with MoO_3_ to form heterostructural photocatalysts that can enhance the interfacial charge transfer and minimize the recombination of photogenerated electron–hole pairs, so that the photocatalytic efficiency is greatly improved.

**Figure 7:**
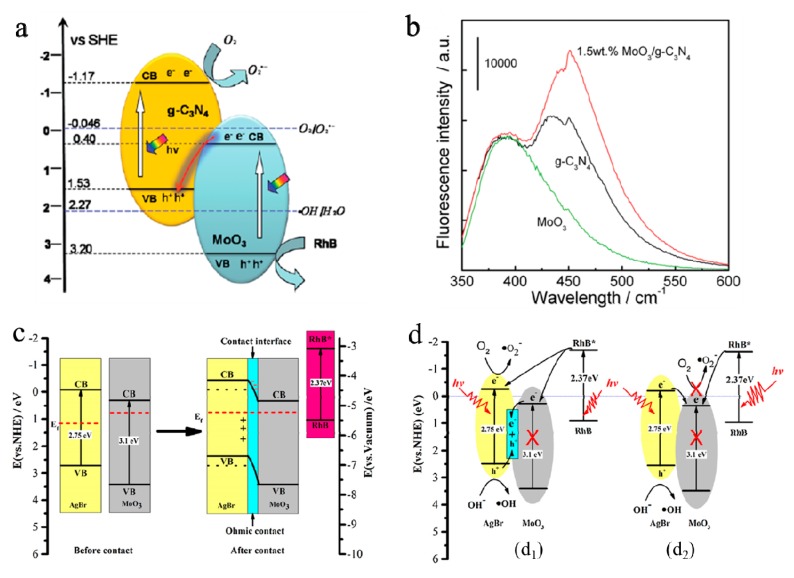
(**a**) Z-scheme mechanism of possible schemes for electron–hole separation and transport at the visible-light-driven MoO_3_–g-C_3_N_4_ composite interface. (**b**) The PL spectra of MoO_3_, g-C_3_N_4_, and 1.5 wt.% MoO_3_–g-C_3_N_4_ samples under visible-light irradiation for 30 min [108]. Reprinted with permission from [108]. Copyright 2014 Royal Society of Chemistry. (**c**) Schematic illustration of photosensitized degradation of the RhB dyes over the AgBr/MoO_3_ composite before and after contact. (**d**) Novel Z-scheme (**d_1_**), and Heterojunction-type (**d_2_**) charge-transfer mechanisms for the AgBr/MoO_3_ system [99]. Reprinted with permission from [99]. Copyright 2017 Elsevier B.V.

Besides, to further shorten degradation period and improve degradation efficiency, the MoO_3_-based direct solid-state Z-scheme system with a visible-light-driven semiconductor photocatalysts have been studied. He et al. [108] developed Z-scheme type MoO_3_–g-C_3_N_4_ for enhanced photodegradation activity of methylene orange under visible light irradiation. The high photocatalytic activity of MoO_3_–g-C_3_N_4_ is mainly attributed to the synergetic effect of MoO_3_ and g-C_3_N_4_ in electron–hole pair separation via the charge migration between the two semiconductors (see Figure 7a). Figure 7b showed the PL spectra of MoO_3_, g-C_3_N_4_ and 1.5 wt.% MoO_3_–g-C_3_N_4_ samples. The 1.5 wt.% MoO_3_–g-C_3_N_4_ sample had the strongest PL peak, the reason was the combination of high concentrations of the electron on the CB of g-C_3_N_4_ and holes on the VB of MoO_3_, which generated higher concentration ·OH species. Feng et al. [99] reported AgBr/MoO_3_ monolithic catalyst for degrading RhB under visible light irradiation. The formation of well-defined novel Z-scheme between AgBr and MoO_3_ effectively accelerated dye-sensitization and charge transfer, resulting in high activity in degrading RhB solution. Figure 7c showed the schematic illustration of photosensitized degradation of the RhB dyes over the AgBr/MoO_3_ composite before and after contacting for the AgBr/MoO_3_ system. Further experiments suggested that ultrafast degradation of the RhB on the AgBr/MoO_3_ nanocomposites was due to both the photocatalytic process and the dye sensitization. The charge-transfer mechanism was shown in Figure 7d. These results opened up a new avenue in surface- and interface-engineering techniques, enhancing the further utilization in the field of energy transformation and environmental improvement.

**Figure 8:**
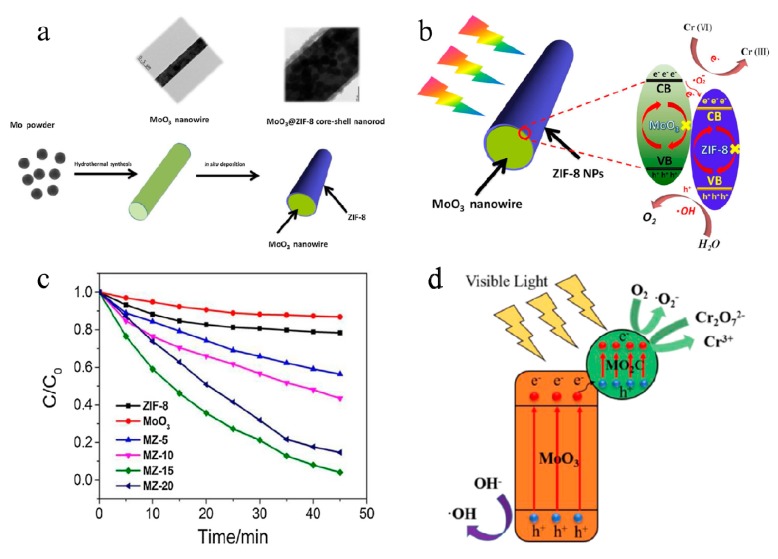
(**a**) Schematic illustration of the synthetic procedure for the hollow MoO_3_@ZIF-8 core–shell nanorods. (**b**) Schematic illustration of the Cr (VI) degradation mechanism. (**c**) Photocatalytic performance of MZ-15 under visible-light irradiation [109]. Reprinted with permission from [109]. Copyright 2018 Elsevier B.V. (**d**) The photocatalytic reaction mechanism of photodegradation of MO and photoreduction of Cr (VI) for Mo_2_C/MoO_3_ under visible-light irradiation [110]. Reprinted with permission from [110]. Copyright 2019 Elsevier.

Apart from the use of decomposing dyes in wastewater, MoO_3_ is also effective for photoreduction of aqueous Cr (VI). Very recently, Zhang et al. [109] prepared three-dimensional (3D) MoO_3_@ZIF-8 core–shell nanorod composite photocatalysts and studied the Cr (VI) degradation mechanism, which were shown in Figure 8a,b. Compared with the pure ZIF-8 and MoO_3_ nanowires, the MoO_3_@ZIF-8 catalysts exhibited superior photocatalytic activity for Cr (VI) reduction under visible light (see Figure 8c). The reduction of Cr (VI) was up to 100% with 15% of ZIF-8 in 45 min. Moreover, the composite had good recyclability, and the photocatalytic activity remained almost constant after four cycles. Recently, Jing et al. [110] designed and synthesized Mo_2_C/MoO_3_ and employed it as catalyst for the photoreduction of Cr (VI) and photodegradation of MO under visible light. The photocatalytic reaction mechanism of photodegradation of MO and photoreduction of Cr (VI) for Mo_2_C/MoO_3_ under visible-light irradiation was illustrated (see Figure 8d). The Mo_2_C/MoO_3_ heterostructure showed the good photocatalytic activity for wastewater treatment.

In brief, MoO_3_ has shown a good activity in degradation of the organic pollutants due to the design of morphology, hybrid structure, and heterostructure in catalysts. However, most photocatalytic processes were finished to decompose dyes under visible-light irradiation, so it is meaningful to synthesize nano-photocatalytic materials with a wide variety of wavelength bands. Additionally, based on the reported works, the study of stability and recyclability is poor; however, it is crucial for the practical applications of catalysts.

### 4.2. Selective Catalysis to Reduce Air Pollutants

#### 4.2.1. Selective Catalytic Reduction of NO_x_ with NH_3_

**Figure 9:**
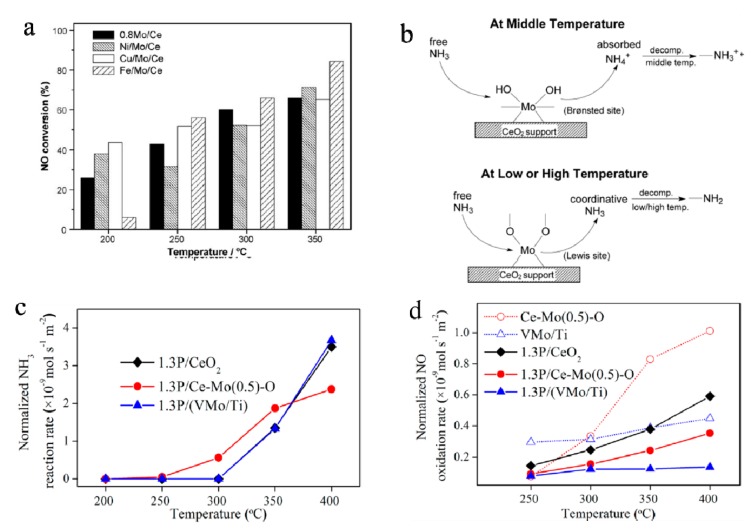
(**a**) The NO conversion for different catalysts in ‘‘NO + NH_3_ + O_2′_’ reaction. (**b**) Schematic drawing of ammonia adsorption and decomposition on Brønsted and Lewis acid sites [116]. Reprinted with permission from [116]. Copyright 2009 Elsevier B.V. (**c**) NH_3_ reaction rate vs. temperature over the Ce–Mo(0.5)–O and CeO_2_ catalysts with different phosphorus content in NH_3_ oxidation reaction. (**d**) Normalized NO oxidation rate vs. temperature in NO oxidation reaction over different catalysts after P poisoning [115]. Reprinted with permission from [115]. Copyright 2013 American Chemical Society.

Nitrogen oxides (NO_x_) cause many environmental pollution problems, such as acid rain, photochemical smog, and ozone depletion. Selective catalytic reduction (SCR) of NO_x_ with NH_3_ has become the dominant technology for decreasing NO_x_ emission from stationary and mobile sources [111,112]. MoO_3_ plays an important role in traditional catalysts, for example, with MoO_3_-based catalysts for SCR exhibiting good catalytic NO_x_ removal performance. The redox behavior of V_2_O_5_–MoO_3_/TiO_2_ catalysts was investigated by Casagrande and co-workers, who pointed out that the ternary catalysts are more easily reduced and reoxidized than the corresponding binary samples (V_2_O_5_/TiO_2_ and MoO_3_/TiO_2_ catalysts) [113]. Recently, based on CeO_2_/MoO_3_ binary or ternary catalysts that were favored by researchers and CeO_2_ that possesses excellent redox property also with plenty of Lewis acid sites, the catalysts with MoO_3_ loaded on CeO_2_ have been well studied, with much more acid sites on surface. The synergistic effect between CeO_2_ and MoO_3_ contributed to the selective oxidation of NH_3_ to N_2_ [114,115]. For instance, Zhu et al. [116] studied the surface structure of M_x_O_y_/MoO_3_/CeO_2_ system (M = Ni, Cu, Fe) and its influence on SCR of NO by NH_3_. The results revealed that the intensity of the interaction between MoO_3_ and other metal oxides was different, which could be listed as follows: NiO/MoO_3_/CeO_2_ > CuO/MoO_3_/CeO_2_ > Fe_2_O_3_/MoO_3_/CeO_2_. The NO conversion for different catalysts in ‘‘NO + NH_3_ + O_2′_’ reaction is shown in Figure 9a. Figure 9b shows the schematic drawing of ammonia adsorption and decomposition on Brønsted and Lewis acid sites. The reactivity of “NO + NH_3_ + O_2_” reaction was strongly associated with acid properties of the catalysts. Additionally, some researchers studied the catalytic behaviors of MoO_3_-based catalysts with respect to resistance to phosphorus, as shown in Figure 9c,d. The 1.3P/Ce–Mo(0.5)–O catalyst showed a higher NH_3_ reaction rate than the other two catalysts below 350 °C, which suggested that the resistance of the CeO_2_ catalyst to phosphate was improved with the addition of Mo, and phosphorus poisoning significantly affected catalyst activity [115]. Nevertheless, the NO_x_ conversion is still within a relatively narrow temperature window for MoO_3_-based NH_3_–SCR catalysts, and it is necessary to develop catalysts with superior catalytic performance at both high and low temperatures, with excellent resistance to coexisting poisoning pollutants such as SO_2_ and phosphate.

#### 4.2.2. Selective Catalytic Oxidation of Propene

**Figure 10:**
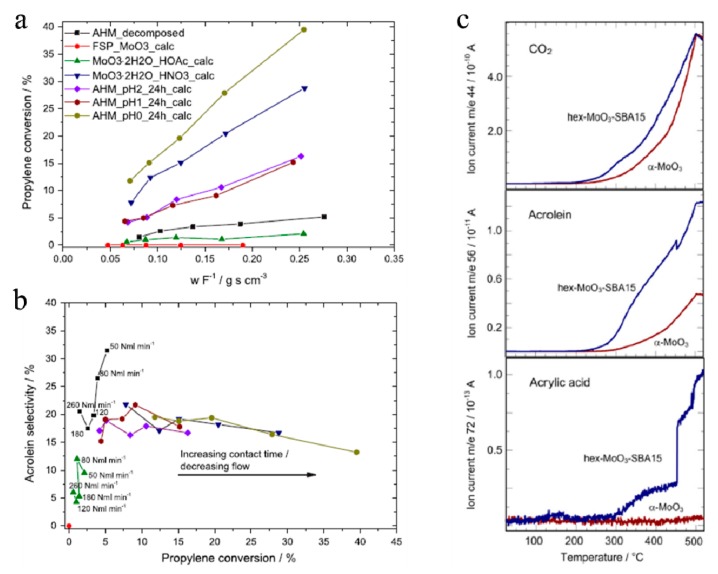
(**a**) The propylene conversion as a function of contact time. (**b**) The acrolein selectivity as a function of propylene conversion [118]. Reprinted with permission from [118]. Copyright 2015 Elsevier B.V. (**c**) Evolution of ion currents of CO_2_, acrolein, and acrylic acid obtained by mass spectrometric analysis of the gas phase composition during thermal treatment of MoO_3_/SiO_2_ or α-MoO_3_ in propene and oxygen [121]. Reprinted with permission from [121]. Copyright 2007 Elsevier B.V.

Propene has an extensive production and application in numerous industries, and it is a primary contributor to photochemical smog, making it unfriendly to the environment [117]. There are lots of reports focusing on the catalytic oxidation of propene. MoO_3_ is well-known for its structure sensitivity in selective oxidation of propene. The products of selective oxidation mainly include acrylic acid and acrolein. Schuh et al. [118] studied the influence of the morphology of α-MoO_3_ in the selective oxidation of propylene. The results suggested that the morphologies of the samples have a significant effect not only on the selectivity to acrolein, but also on the propylene conversion (see Figure 10a,b). The rod-like structures with (100) facet seemed to be of decisive importance for the catalytic activity, and the same conclusion was drawn by Volta and coworkers [119,120]. Moreover, supported Mo oxides may exhibit different structural and catalytic properties for selective oxidation of propene. Ressler et al. [121] systematically studied the catalytic properties of MoO_3_ supported on nanostructured SiO_2_ and compared the conversion quantity of h-MoO_3_/SiO_2_ with that of α-MoO_3_ by mass spectrometric analysis of the gas-phase composition during thermal treatment of in propene and oxygen (see Figure 10c). It exhibited stability and catalytic properties different from other binary bulk oxides and could directly oxidize propene to acrylic acid without additional metal sites. Besides this, MoO_3_ was used for the epoxidation of propylene to propylene oxide (PO) by molecular oxygen [122]. The work of Jin et al. [123,124] on Ag–MoO_3_ and Ag–MoO_3_–ZrO_2_ catalysts showed that MoO_3_ resulted in a significant improvement in the efficiency of the catalyst. The addition of MoO_3_ increased the selectivity to 34% at a propylene conversion of 4.6%, whereas the pure Ag catalyst had a PO selectivity of only 0.8% at a propylene conversion of 11%. The olefinic carbon of propylene was easily adsorbed by adding MoO_3_ into the silver catalyst. MoO_3_ was as an electron and structure-type bifunctional promoter in this system.

#### 4.2.3. Selective Catalytic Oxidation of Methane

As one of the greenhouse gases, methane effects on global warming. Hence, its emissions have attracted more and more attention [125]. The conversion of CH_4_ into chemicals is much more economical and energy-efficient compared to CO_2_. Many publications in this field have been totally committed to the oxidation of methane at relatively high temperature, using transition metal oxides catalysts. The catalysts based on MoO_3_ were the most widely studied because of their higher activity and selectivity for methane oxidation [126]. Liu et al. [127] prepared MoO_3_/SiO_2_ catalysts and studied the kinetics and mechanism of the partial oxidation of methane, using N_2_O as the oxidant. These researchers also found that the selective oxidation reaction was initiated by the formation of O^−^ ions generated from the interaction of N_2_O. The structural effects of MoO_3_ on partial oxidation of methane to formaldehyde were investigated by Smith et al. The results indicated that Mo=O sites located on the side plane tend to form formaldehyde [128]. Moreover, Arena et al. [129] studied the working mechanism of MoO_3_/SiO_2_ catalysts in the partial oxidation of methane to Formaldehyde. The influence of the oxide loading on the surface structure and compared with the dispersity of MoO_3_/SiO_2_ and V_2_O_5_/SiO_2_ catalysts. Taylor et al. [130] prepared a series of catalysts based on MoO_3_ and WO_3_, the MoO_3_ based catalysts were more effective for the production of methanol. The Cu/MoO_3_ and Ga_2_O_3_/MoO_3_ catalysts showed selectivity and methanol yield advantage, respectively. MoO_3_ demonstrated oxygen insertion ability due to its n-type semiconductivity. However, the mechanism of the co-operative effect in catalysis was not well studied. Most studies focused on the catalytic activity and selectivity, but the research of durability is rather poor.

#### 4.2.4. Other Catalysis to Reduce Air Pollutants

There are only a few reports about MoO_3_ catalysts applied for oxidation of CO and some volatile organic compounds (VOCs), such as methanol, (CH_3_)_2_S_2_, benzene, and chlorobenzene [131,132,133]. Mohamed et al. [131] reported the oxidation of CO to CO_2_ by MoO_3_/CeO_2_, and the results suggested that the dispersed MoO_x_ species and Ce^3+^/Ce^4+^ redox couples had high capacity toward oxygen, which was most likely to be the active species for CO oxidation. Wang et al. [132] studied the catalytic incineration of (CH_3_)_2_S_2_ on CuO–MoO_3_/γ-Al_2_O_3_ and the promoter effect. The results revealed that CuO–MoO_3_/γ-Al_2_O_3_ has a good activity and durability, and Cr_2_O_3_ was the most effective promoter. Moreover, MoO_3_ as a dopant in V_2_O_5_–MoO_3_/TiO_2_ catalysts improved redox properties and enhanced chlorobenzene oxidation catalytic activity at a low temperature, and this illuminated new applications for pollutants [133]. These researches will lay an early foundation for exploring MoO_3_ catalytic properties in the catalysis of other VOCs.

## 5. Summary and Outlook

Nanostructured MoO_3_ with unique structures and wide availabilities become one of the most promising materials employed for versatile applications. In this review, we have presented the basic structure and main applications of nanostructured MoO_3_. The MoO_3_ mainly consists of four phases: orthorhombic (α), monoclinic (β), hexagonal (h), and high pressure monoclinic (ε). All of these phases display unique physical and chemical properties for different performances.

We mainly outlined the applications of nanostructured MoO_3_ in the field of energy and environmental catalysis, including water splitting, fuel cells, photocatalytic degradation, and selective thermocatalysis. The catalytic activities of nanostructured MoO_3_ will be improved through the design of morphology, hybridization, and hybrid structure. The bandgap structure is optimized, and charge transfer is enhanced by constructing heterojunction nanocomposites. Although progress has been made in catalysis based on nanostructured MoO_3_, the current research is at nascent stage. It is still required to develop new nanostructured MoO_3_ materials to meet more demands in practical applications. For instance, selective catalysis to remove air pollutants requires very high temperatures; therefore, the exploitation of new and improved nanostructured MoO_3_ applied to mild conditions is needed. The catalytic nature and mechanism in MoO_3_-based catalysts are still challenging and need further studies. Limited research has been done to investigate the stability and recyclability in the selective thermocatalysis. There are numerous research studies about MoO_3_ materials used for energy catalysis, especially in the field of hydrogen evolution. All kinds of strategies, including (1) morphology control, (2) phase transition, (3) introduction of oxygen vacancies, (4) hetero-atom doping, and (5) hybridization with other materials, have been employed to improve catalytic performance and durability of MoO_3_-based materials. However, the activity or performance of MoO_3_-based materials still do not completely reach the commercial standard, and the mechanism is not fully understood. The strategies mentioned above have been carried out, but there is no systematic and deep study on morphology, phase, oxygen vacancies, etc. Therefore, many problems, such as ‘how do oxygen vacancies influence catalytic performance?’ still need to be solved. It is more common to see that MoO_3_ serves as a support material instead of active centers as a whole. Although some researchers have devoted themselves to addressing this [55,61,62,63,64,65], the activity enhancement is still limited. On the other hand, the durability of MoO_3_-based materials still needs to be improved, and the stability test time should be prolonged. Thus, MoO_3_ has huge treasure to be excavated for HER, OER, and fuel cells. In short summary, generally speaking, there are two aspects for further studying MoO_3_ in the future: (i) the great improvement of catalytic performance and stability; and (ii) insight into structure-activity relationship and mechanism. Novel strategies and new methods must be proposed in order to achieve these long-term goals.
